# Evaluation of toothpastes for treating root carious lesions – a laboratory-based pilot study

**DOI:** 10.1186/s12903-024-04061-8

**Published:** 2024-04-22

**Authors:** Haoran Chen, Jiaxin Zhang, Robert Hill, Aylin Baysan

**Affiliations:** 1 Institute of Dentistry, Barts and The London School of Medicine and Dentistry, Queen Mary University of London, London, UK; 2 Biochemical Pharmacology, William Harvey Research Institute, Queen Mary University of London, London, UK

**Keywords:** Bioactive glass, Artificial root caries, Toothpastes, Fluoride, Fluorapatite

## Abstract

**Background:**

Root caries is preventable and can be arrested at any stage of disease development. The aim of this study was to investigate the potential mineral exchange and fluorapatite formation within artificial root carious lesions (ARCLs) using different toothpastes containing 5,000 ppm F, 1,450 ppm F or bioactive glass (BG) with 540 ppm F.

**Materials and methods:**

The crowns of each extracted sound tooth were removed. The remaining roots were divided into four parts (*n* = 12). Each sample was randomly allocated into one of four groups: Group 1 (Deionised water); Group 2 (BG with 540 ppm F); Group 3 (1,450 ppm F) and Group 4 (5,000 ppm F). ARCLs were developed using demineralisation solution (pH 4.8). The samples were then pH-cycled in 13 days using demineralisation solution (6 h) and remineralisation solution (pH 7) (16 h). Standard tooth brushing was carried out twice a day with the assigned toothpaste. X-ray Microtomography (XMT) was performed for each sample at baseline, following ARCL formation and after 13-day pH-cycling. Scanning Electron Microscope (SEM) and ^19^F Magic angle spinning nuclear magnetic resonance (^19^F-MAS-NMR) were also performed.

**Results:**

XMT results showed that the highest mineral content increase (mean ± SD) was Group 4 (0.09 ± 0.05), whilst the mineral content decreased in Group 1 (-0.08 ± 0.06) after 13-day pH-cycling, however there was evidence of mineral loss within the subsurface for Groups 1, 3 and 4 (*p* < 0.05). SEM scans showed that mineral contents within the surface of dentine tubules were high in comparison to the subsurface in all toothpaste groups. There was evidence of dentine tubules being either partially or completely occluded in toothpaste groups. ^19^F-MAS-NMR showed peaks between − 103 and − 104ppm corresponding to fluorapatite formation in Groups 3 and 4.

**Conclusion:**

Within the limitation of this laboratory-based study, all toothpastes were potentially effective to increase the mineral density of artificial root caries on the surface, however there was evidence of mineral loss within the subsurface for Groups 1, 3 and 4.

## Introduction

The World Health Organisation recently reported that one in six people will be over 60 years old by 2030 and by 2050, this population will double. Individuals aged 80 years or older are expected to triple between 2020 and 2050 to reach 426 million [1].

Root caries is one of the major and preventable global oral health problems in aging population [2]. Root surfaces are more susceptible to demineralisation in acidic conditions than enamel. High levels of carbonate and magnesium in the apatite, large exposed surface areas and dentine tubular structure can result in increased incidence of root cares [3].

The conventional approach of ’drilling and filling’ remains clinically challenging due to the high organic content of root dentine, close proximity to both the gingivae and dental pulp, difficulty in obtaining direct access in some cases and moisture control [4]. Therefore, it is imperative to establish cost-effective and efficient management strategies for root caries [5]. In this respect, early detection and minimally invasive techniques for treating root caries have previously been proposed [6]. The caries preventive effect of fluoride is related to inhibiting demineralisation and promoting remineralisation by minimising the solubilisation of the apatite crystals during periods of pH decrease and facilitating the remineralisation of partially dissolved crystals through pH cycling [7]. Fluoridated toothpastes are regarded as effective fluoride delivery vehicles [4]. It was reported that over 95% of the population in developed countries use fluoridated toothpastes [8].

In this respect, Baysan et al., (2001) previously demonstrated that toothpaste containing 5,000 ppm F was capable of reversing 57% of leathery primary root carious lesions to hard lesions. In comparison toothpaste containing 1,100 ppm F reversed 29% [9]. Subsequently, Ekstrand et al., (2008) indicated that the high fluoridated toothpaste significantly improved the management of root caries in comparison to the 1,450 ppm F [10]. It should be noted that fluoride levels in saliva and biofilms decrease significantly within 15 min following the use of fluoridated toothpaste and rinsing. The presence of fluoride reservoirs can be recharged with the use of fluoridated toothpaste. These reservoirs would then slowly release fluoride ions [11]. Therefore, bioavailable fluoride is beneficial to provide a favourable impact on promoting remineralisation and inhibiting demineralisation [8, 12].

Bioactive glass (BG) with low fluoride and high phosphate content was recently incorporated into toothpastes. These amorphous silicate glasses degrade in aqueous solutions [13] and release calcium, phosphate and fluoride ions. This process can then promote fluorapatite formation [14]. Recent studies reported that fluoride ions released from the fluoride containing BGs were retained up to one week [14, 15]. Naumova et al., (2019) also reported that there was a positive effect on bioavailable fluoride following the use of toothpaste containing fluoride and BG [13]. These indicated the increased concentration of fluoride ions in oral cavity, which would contribute to the remineralisation process. In addition, Bakry et al., (2018) demonstrated that the same toothpaste was effective in treating subsurface enamel lesions in vitro [16]. The previous study showed toothpaste containing fluoride and BG can promote fluorapatite formation in root carious lesions [17]. However, these studies have not investigated any mineral exchange within root caries following the use of toothpaste containing either sodium fluoride or low concentration fluoride in bioactive glasses. Therefore, the aim of this pilot laboratory-based study was to investigate the potential mineral exchange and fluoridated apatite formation within the subsurface of artificial root carious lesions (ARCLs) following the use of fluoridated toothpastes with or without bioactive glass.

## Materials and methods

### Sample preparation

Three extracted sound teeth without dental caries, sealant, restorations or cracks were collected for the study from the Oral Surgery. Ethical approval was obtained prior to the study (QMREC 2011/99). Each tooth was stored in 0.1% thymol [18]. These teeth were then carefully cleaned and polished using non-fluoridated prophylaxis paste (NUPRO Dentsply, USA). The crowns of each tooth were removed 1 mm below the cemento-enamel junction by cutting the root surfaces using a 0.3 mm thick diamond disc under running water at 3,000 rpm speed (Struers, Copenhagen, Denmark) and divided into four sections in order to reduce any potential tooth to tooth variations. Subsequently, these four samples were allocated into each group (Table 1).

**Table 1:** The allocated study groups

Groups	Toothpastes	Name Company	Ingredients
Group 1	Deionised water	Avidity Science, UK	
Group 2	BG with 540 ppm F	BiominF, Biomin®, UK	Glycerin, Silica, PEG400, Fluoro Calcium Phospho Silicate, Sodium lauryl Sulphate, Titanium dioxide, Aroma, Carbomer, Potassium Acesulfame
Group 3	1,450 ppm NaF	Colgate-Advanced White, Colgate, UK	Sodium Fluoride, Aqua, Hydrated Silica, Sorbitol, PEG-12, Sodium bicarbonate, Aroma, Sodium lauryl sulfate, Xanthan gum, Cellulose gum, Sodium saccharin, Limonene, CI 74,160, CI 77,891
Group 4	5,000 ppm NaF	Duraphat®, Colgate, UK	Sodium Fluoride, Liquid Sorbitol (Non-crystallising), Silica, Silica (precipitated), Macrogol 600, Tetrapotassium Pyrophosphate, Xanthan Gum, Sodium Benzoate, Sodium lauryl sulfate, Spearmint Oil, Peppermint Oil, Carvone, Menthol, Anethol, Lemon Oil, Saccharin Sodium, Brilliant Blue FCF, Purified Water

### Formation of artificial root carious lesions

Lesions were formed in the 1.5 mmol/L CaCl_2_, 0.9 mmol/L KH_2_PO_4_, 50 mmol/L acetic acid and 1 M KOH adjusted to pH 4.8 for five days. In addition, 5.0 mmol/L NaN_3_ was added to the solution to prevent microbial growth. Each sample was separately placed in 10 ml of the solution with new solution changed every single day. During this period, these samples were kept in a 60 rpm shaking incubator (Staufen, Germany) at 37 °C. In the meantime, the procedure avoided any possible desiccation to prevent the shrinkage of ARCLs [19, 20]. .

### The pH-cycling conditions

The rationale of the pH-cycling process was to simulate the dynamics between mineral loss and uptake as seen in the formation of dental caries [21]. This process contained exposure of dentine to alternate demineralisation and remineralisation cycles. The demineralisation solution composition was described in the preparation of artificial root carious lesions.

The remineralisation solution contained 1.5 mmol/L CaCl_2_, 0.9 mmol/L KH_2_PO_4_, 20 mmol/L HEPES and 130 mmol/L KCl. 5.0 mmol/L NaN_3_ was added to the solution to prevent microbial growth. The pH was adjusted to 7.0 using 1 M KOH. All chemical reagents were obtained from Sigma Aldrich, UK. Each sample was immersed in 10 mL of the demineralisation solution for six hours followed by immersion in 10mL of the remineralisation solution for 16 h. Fresh solutions were used for each cycle. This procedure was performed for a period of 13 days at 37 °C [20, 22].

### The use of different toothpastes

A total of 12 samples with ARCLs were allocated into four different treatment groups. Each group (*n* = 3) received one of the allocated treatments (Table 1). Each toothpaste was diluted with deionised water at a dilution of 1:3 to make a toothpaste slurry. Tooth brushing was simulated using a medium-bristle toothbrush (Colgate Palmolive, UK) twice a day for 13 days. The brushing process was carried out using an electrically-powered tooth brushing machine (Boston Gear, Braintree, MA) designed to produce constant reciprocal movements with 150 g force for 10 s [23]. The samples were left for two minutes *prior* to rinsing with deionised water [24].

### Non-destructive X-ray microtomography (XMT)

The assessment of mineral density was carried out by the MuCAT XMT scanner. The in-house developed system was designed by means of time-delay integration to provide high quality tomographic images. Each sample was separately placed in a polymethyl methacrylate (PMMA) mould. Subsequently, the mould with samples was put inside a clear plastic tube with a drop of deionised water to keep the specimen fully hydrated and avoid contamination during the long scanning procedure (around 24 h). The prepared sample was then mounted onto the XMT movable kinematic stage, ensuring the long axis of the tooth was parallel to the XMT rotational axis. The XMT scanner was set at 15 μm voxel size resolution (3D). The x-ray generator was operated at 90 kV and 270 µA. After each sample scan, a calibration was performed and the projection data was transformed to 30 keV monochromatic energy equivalent [25]. The reconstructed linear attenuation coefficients (LACs) were converted to the level of mineral density [24]. The XMT scans were carried out at baseline, following the development of ARCLs, and after 13 days of pH-cycling for each sample.

### XMT Image analysis

**Fig. 1:**
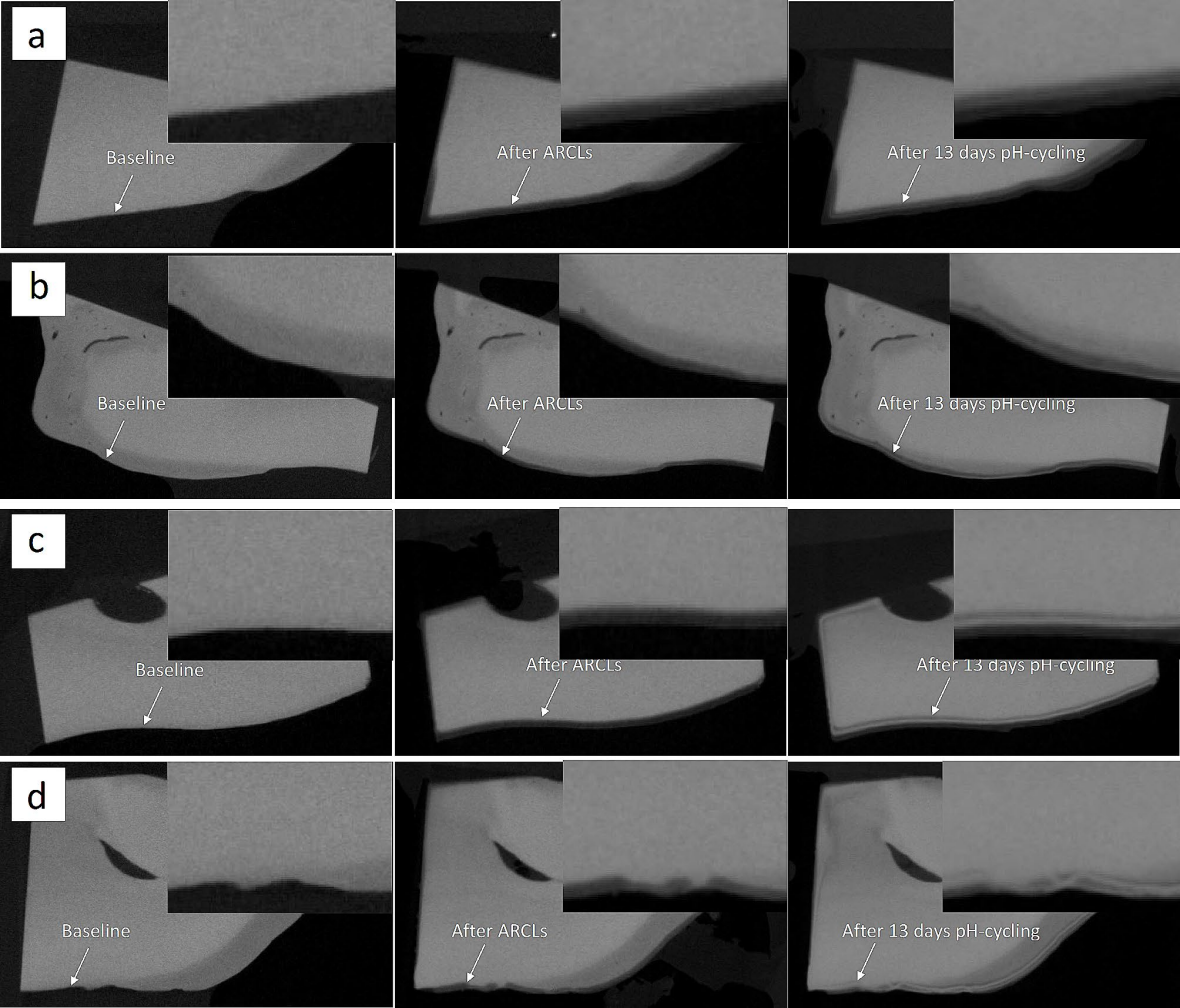
Part of a single slice from the re-constructed XMT image of a sample treated by **(a)** Group (1) Deionised water; **(b)** Group (2) BG with 540 ppm F; **(c)** Group (3) 1,450 ppm F; **(d)** Group (4) 5,000 ppm F; (left) baseline, (middle) after the development of ARCLs, (right) after the application of different treatments with 13 days of pH-cycling

The 3D images from the baseline and treatment scans after 13 days of pH-cycling were aligned using an in-house developed software called ‘Tomview’. These 3D images were then subtracted by running the International Data Line (IDL, Exelis Visual Information Solutions, Boulder, Colorado, USA) software and subsequently mineral loss/gain was detected according to the increase in radiopacity/radiolucency in detected areas (Fig. 1a-d). Randomly selected 45 points of LACs in these detected areas after each ARCL scan were compared with the corresponding 45 points in the baseline scans, and also with the treatment scans after 13 days of pH-cycling [24]. Each line profile was also conducted using ImageJ (U. S. National Institutes of Health, Bethesda, Maryland, USA). A line profile was drawn through the low mineral density to the high mineral density parts. These line profiles were then recorded for the successive scans by clicking on the plugins and macros, then recording the sections. This process was carried out to ensure that the line profile, which was drawn, was exactly in the same position for all the successive scans for that section of each sample. The line profile of all samples in each group was cumulated average to avoid the tooth variation.

The obtained LAC values were then converted to mineral density (g cm^− 3^) using the following equation:

$$c = \frac{{\mu - \mu 0}}{{\mu m - \mu 0}}\rho m$$

Where μ is the measured LAC, μ0 is the LAC of the deionised water and plastic that assumed soft tissue (0.268 cm-1), μm is the pure sample material LAC that presumed pure hydroxyapatite (3.12 cm^-1^), and *ρm* is the concentration of pure hydroxyapatite (3.16 cm^-3^).

### Scanning Electron microscope (SEM)

This surface morphology of each sample was observed with scanning electron microscopy (SEM, FEI Inspect-F, Hillsboro, USA) after coating with carbon nanofilm (∼ 30 nm) using the sputter coating machine (SC 7620, Quorum, Laughton, UK) to maintain conductivity, while the samples were analysed at an accelerating voltage of 5 kV, with a spot size of 3.0 and variable working distances (around 10 mm) for secondary electrons (SE). Subsequently, the voltage increased by 20 kV with a spot size of 5.0 for backscattered electron (BSE) imaging.

### ^**19**^**F Magic angle spinning nuclear magnetic resonance (MAS-NMR)**

The ^19^F-MAS-NMR enables fluorapatite to be distinguished from hydroxyapatite. ^19^F-MAS-NMR were carried out using a 600 MHz Bruker spectrometer with a magnetic field of 14.1 Tesla. The ^19^F-MAS-NMR spectra were collected at the resonance frequency of 564.7 MHz. The samples were run in a 2.5 mm zirconia rotor spinning at 22 kHz using the standard double resonance Bruker probe with low fluorine background. 1 M aqueous solution of sodium fluoride producing a sharp signal – 120 ppm was used to reference at the ^19^F chemical shift scale. This was run with a 30 s recycle delay with 1 µs pulse at 100 W.

### Statistical analysis

The percentages in mineral density changes were calculated. The paired *t*-test was then carried out to compare the differences in mineral contents for each sample in the intra-groups. The data from XMT mineral density in inter-groups comparison was analysed using the Kruskal-Wallis H with Mann-Whitney test nonparametric tests due to non-normal distribution. A significance level of 0.05 was performed using IBM SPSS Statistics 28.0 (SPSS Inc., Chicago, IL, USA).

## Results

### Mineral density using XMT

The quantitative mineral density surface measurements (Table 2) demonstrated an increase in mineral contents within the ARCL areas for each toothpaste group after 13 days of pH-cycling. The surface mineral density (mean ± SD) increased for Group 2 (0.11 ± 0.10), Group 3 (0.30 ± 0.07) and Group 4 (0.22 ± 0.10), whilst there was an evidence of decrease in the Group 1 (-0.07 ± 0.10) (*p* < 0.05).

**Table 2:** The mineral density (g cm^− 3^) in the lesion area at baseline, after the development of ARCLs, and following 13 days pH-cycling (± SD), mean differences and *p*-value, with the percentage of mineral change for all groups at the tooth surface

Groups	Root dentine (baseline)	After ARCLs	13 days of pH-cycling	Mean difference	*p*-value	Overall change	*p*-value (intergroups)
Group 1. Deionised water	1.04 ± 0.34	0.75 ± 0.30	0.63 ± 0.18	-0.12 ± 0.27	0.003	-0.07 ± 0.10 (7% decrease)	Kruskal-Wallis H, H [3] = 125.310, *p* < 0.001, all intergroups comparison (*p* < 0.001)
	1.48 ± 0.19	0.51 ± 0.15	0.42 ± 0.13	-0.09 ± 0.05	< 0.001		
	1.33 ± 0.08	0.73 ± 0.19	0.73 ± 0.11	0.003 ± 0.16	0.006		
Group 2. BG with 540 ppm	1.32 ± 0.10	0.85 ± 0.24	0.80 ± 0.13	-0.05 ± 0.17	0.130	0.11 ± 0.10 (11% increase)	
	1.26 ± 0.18	0.77 ± 0.21	0.84 ± 0.08	0.07 ± 0.18	0.010		
	1.20 ± 0.18	0.66 ± 0.16	0.96 ± 0.12	0.30 ± 0.15	< 0.001		
Group 3. 1,450 ppm	1.21 ± 0.12	0.83 ± 0.19	0.96 ± 0.10	0.13 ± 0.18	< 0.001	0.30 ± 0.07 (30% increase)	
	1.38 ± 0.11	0.45 ± 0.13	0.86 ± 0.24	0.41 ± 0.13	< 0.001		
	1.33 ± 0.07	0.64 ± 0.06	0.99 ± 0.07	0.35 ± 0.05	< 0.001		
Group 4. 5,000 ppm	1.24 ± 0.17	1.04 ± 0.22	1.08 ± 0.22	0.04 ± 0.20	0.003	0.22 ± 0.10 (22% increase)	
	1.26 ± 0.13	0.71 ± 0.17	1.04 ± 0.13	0.33 ± 0.17	< 0.001		
	1.26 ± 0.20	0.72 ± 0.13	1.00 ± 0.18	0.28 ± 0.10	0.006		

### Line profile of mineral density by XMT

**Fig. 2:**
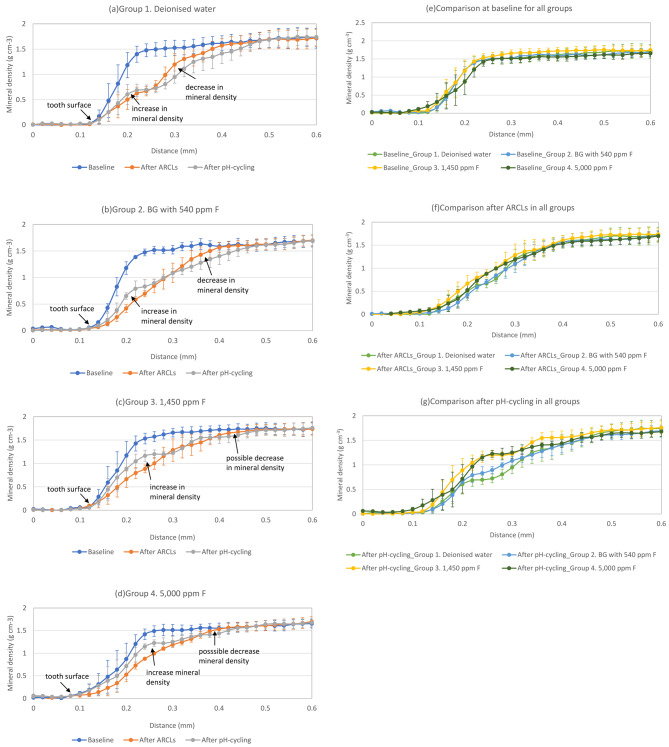
Line profile plotted between varying mineral density (g cm^− 3^) and distance(mm) in the root dentine from surface to subsurface. **(a)** Group (1) Deionised water; **(b)** Group (2) BG with 540 ppm F; **(c)** Group (3) 1,450 ppm F; **(d)** Group (4) 5,000 ppm F; **(e)** Comparisons at baseline for all groups; **(f)** Comparisons after ARCLs in all groups; **(g)** Comparison after pH-cycling in all groups

A line profile through all the ARCLs showed evidence of mineral loss compared to the baseline measurements. In addition, after 13 days of pH-cycling, there was an increase in the mineral density on the surface, whilst a decreased mineral density was observed within the subsurface for all groups (Fig. 2).

The mineral density in Group 1 increased 0.14 mm from the surface towards subsurface. There was a decreased in mineral density from 0.26 to 0.5 mm after the pH-cycling (Fig. 2a). In Group 2, the mineral density increased 0.18 mm in depth from the surface to the subsurface after the pH-cycling. However, mineral loss was noticeable between 0.30 and 0.50 mm from surface (Fig. 2b).

In the Group 3, the total mineral density changes from the surface to subsurface was 0.4 mm, and there was an increased in mineral density from 0.12 to 0.28 mm and 0.34 to 0.38 mm from the surface to subsurface after the pH-cycling (Fig. 2c). The loss of minerals also occurred in the deep subsurface from 0.30 to 0.32 mm and 0.38 to 0.5 mm.

Finally, the depth of increased mineral density after pH-cycling was 0.28 mm in the Group 4, whilst the mineral loss was seen between 0.36 and 0.44 mm from surface (Fig. 2d).

The comparison for all groups in the baseline showed that the mineral density differences were not obvious from sound root dentine (Fig. 2e). The ARCLs comparison in all groups was presented an obvious mineral density loss and the mineral density change keep consistent (Fig. 2f). After pH-cycling with toothpastes treatment, the mineral density for Group 2–4 was higher than that in the Group 1 (Fig. 2g).

The change in mineral density was calculated by the quantitative analysis of the line profiles from the surface to subsurface, which are presented in Table 3. The increased mineral density (mean ± SD) from the surface to subsurface was high in the Group 3 (0.12 ± 0.10) and Group 4 (0.13 ± 0.08) when compared to that in the Group 2 (0.10 ± 0.08). The decreased mineral density was similar in the subsurface for Groups 3 and 4 (*p* < 0.05).

**Table 3:** The relative mineral density (g cm^− 3^) changes (SUM^*^; mean ± SD) from the plotted line profiles from Fig. 2 at the tooth subsurface. (SUM^*^ is the integrity mineral density change)

Groups	Baseline-after ARCLs			ARCLs-After 13 days of pH-cycling								
	Decrease		*p*-value	Increase		*p*-value	Subsurface decrease		*p*-value	Net change in mineral content		
	SUM^*^	mean ± SD	Kruskal-Wallis H, H [3] = 0.834, *p* = 0.841; intergroup comparison: Mann-Whitney test: Groups 1 and 2 (*p* = 0.978), Groups 1 and 3 (*p* = 0.705), Groups 1 and 4 (*p* = 0.626), Groups 2 and 3 (*p* = 0.871), Groups 2 and 4 (*p* = 0.516), Groups 3 and 4 (*p* = 0.358)	SUM^*^	mean ± SD	Kruskal-Wallis H, H [3] = 6.435, *p* = 0.092; intergroup comparison: Mann-Whitney test: Groups 1 and 2 (*p* = 1.131), Groups 1 and 3 (*p* = 0.037), Groups 1 and 4 (*p* = 0.020), Groups 2 and 3 (*p* = 0.468), Groups 2 and 4 (*p* = 0.375), Groups 3 and 4 (*p* = 0.922)	SUM^*^	mean ± SD	Kruskal-Wallis H, H [3] = 8.028, *p* = 0.045; intergroup comparison: Mann-Whitney test: Groups 1 and 2 (*p* = 0.311), Groups 1 and 3 (*p* = 0.021), Groups 1 and 4 (*p* = 0.044), Groups 2 and 3 (*p* = 0.087), Groups 2 and 4 (*p* = 0.191), Groups 3 and 4 (*p* = 0.724)	SUM^*^		mean ± SD
Group 1. Deionised water	5.43	0.27 ± 0.28		0.22	0.04 ± 0.04		1.52	0.12 ± 0.07		-1.30	-0.08 ± 0.06	
Group 2. BG with 540 ppm F	5.93	0.30 ± 0.30		0.88	0.10 ± 0.08		0.98	0.09 ± 0.06		-0.10	0.01 ± 0.07	
Group 3. 1,450 ppm F	5.25	0.26 ± 0.21		1.47	0.12 ± 0.10		0.34	0.04 ± 0.03		1.13	0.08 ± 0.06	
Group 4. 5,000 ppm F	4.17	0.21 ± 0.18		1.79	0.13 ± 0.08		0.28	0.04 ± 0.03		1.51	0.09 ± 0.05	

### SEM analysis

**Fig. 3:**
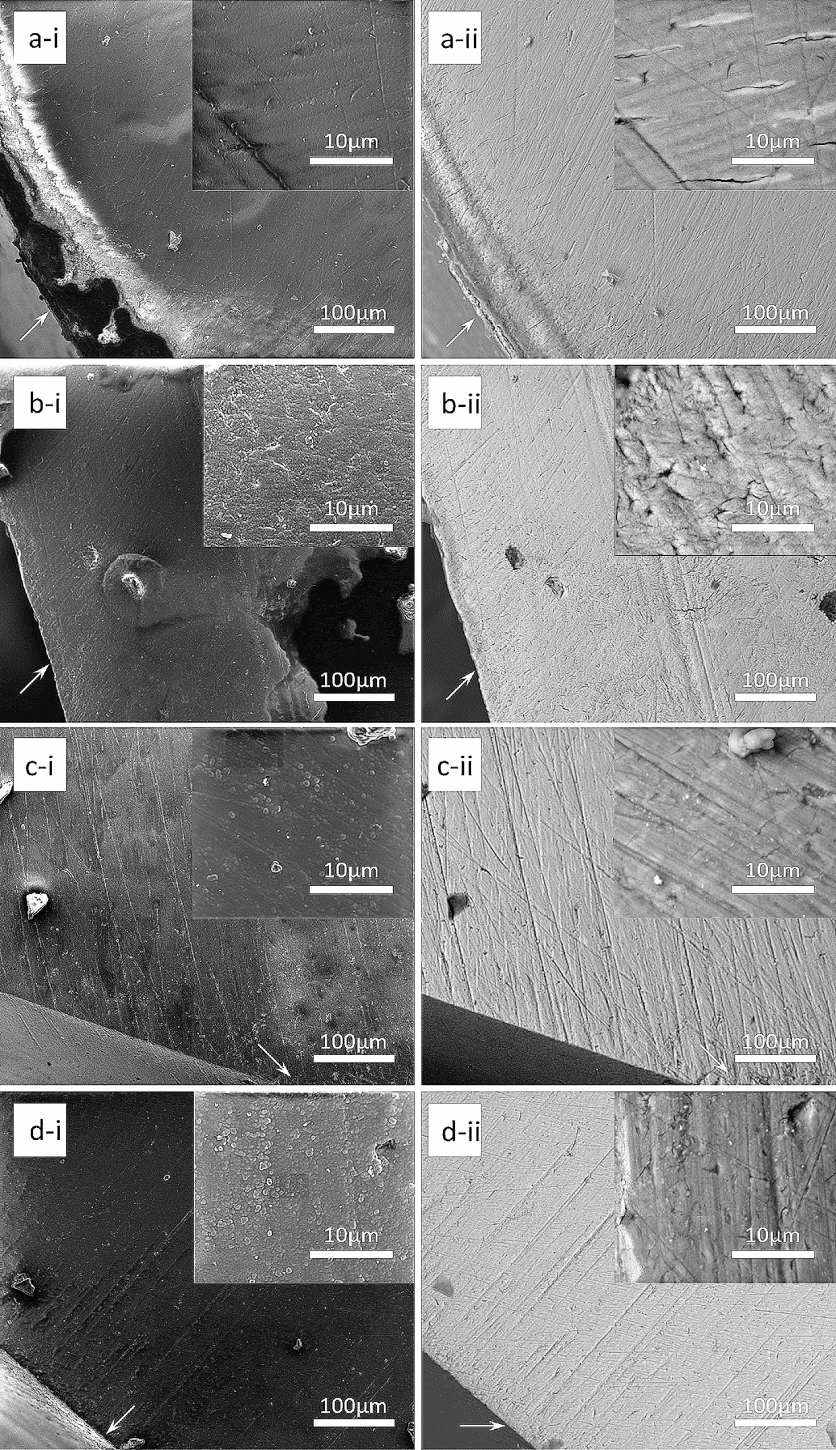
Representative SEM images of SE and BSE (500x and 5000x) of each sample after 13 days of pH-cycling; sample treated with deionised water in SE (a-i) and BSE **(a-ii)**; sample treated with toothpaste containing BG with 540 ppm F in SE **(b-i)** and BSE **(b-ii)**; sample treated with toothpaste containing 1,450 ppm F in SE **(c-i)** and BSE **(c-ii)**; sample treated with toothpaste containing 5,000 ppm F in SE **(d-i)** and BSE **(d-ii)**

The SEM images showed that the dentine tubules were aligned parallel within the subsurface structure of ARCLs. The surfaces were rough and irregular as seen in the following images (Fig. 3a-i). The mineral loss was observed on the ARCL surface in the Group 1 (Fig. 3a-ii).

In addition, the mineral contents within the surface of the dentine tubules were high in comparison to the subsurface in all toothpaste groups. There was also evidence of dentine tubules being either partially or completely occluded in the toothpaste groups.

### ^**19**^**F MAS-NMR**

**Fig. 4:**
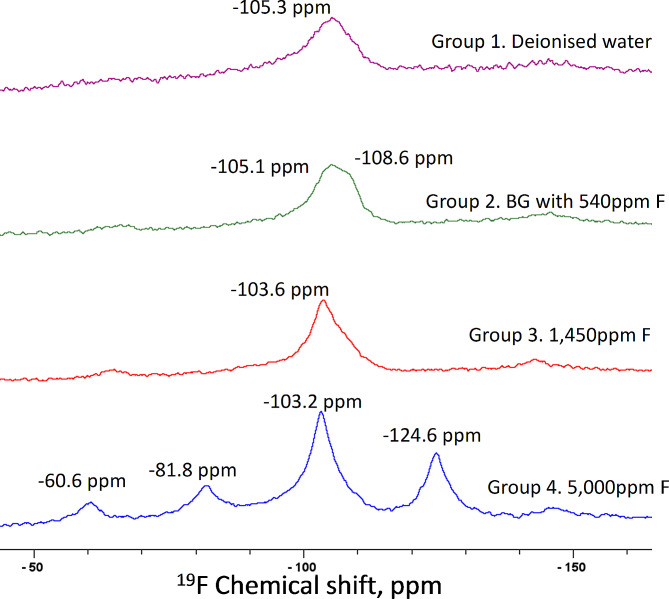
^19^F MAS NMR of samples after 13-days pH-cycling: Group 1. Deionised water; Group-2. BG with 540 ppm F; Group 3. 1,450 ppm F; Group 4. 5,000 ppm F

The spectra of Group 1 (-105.3 ppm) and Group 2 (-105.1 ppm) corresponded to a partial fluoride substituted apatite. An additional peak in the spectra for Group 2 at -108.6 ppm corresponding to the signal of fluoride in crystalline calcium fluoride (CaF_2_) was reported. However, this could be an artefact. The spectra of Groups 3 and 4 indicated the dominant presence of the fluorine-19 signal at -103.6 ppm and − 103.2 ppm respectively (Fig. 4). The strong spinning sidebands were also observed in the ^19^F MAS-NMR spectra for Group 4.

## Discussion

There is limited evidence on the effect of different toothpastes containing either sodium fluoride or low concentration fluoride in bioactive glasses on root caries. To simulate the caries process, artificial saliva rather than simulated body fluid was used in this study. The immersion solution was changed at each tooth brushing time point to simulate the effect of saliva exchange under the in vivo conditions. Furthermore, each sample for the study groups was derived from the same tooth. Interestingly, the amount of mineral density was different from the baseline to ARCLs in each group (Table 3). This means that there were variations in different parts of the same tooth.

The mineral density increases at the surface of ARCLs in Groups 1–3 demonstrated the ability of toothpastes to precipitate minerals and potentially remineralise the surface of ARCLs. This outcome directly corresponded to the surface mineral density changes in the line profiles. The mineral density increase was also observed within the lesion surface in all three toothpaste groups, which supported the previous study [24]. The study reported an increase of 22% in the mineral density by using the 1,450ppm fluoride toothpaste with remineralisation solution alone for a period of 45 days [24]. The pH-cycling model was designed to evaluate the effect of fluoride in either reducing demineralisation or enhancing remineralisation [26]. Chen et al., (2023) showed the significant increase of hardness within root carious lesions following the use of toothpaste treatment with 13 days pH-cycling. Regarding the Knoop hardness, the penetration was up to 0.1 mm depth, which was lower than the real mineral density change (0.4 mm depth). Although the BG with 540 ppm F was proven to show the highest hardness in all toothpaste groups, the mineral density was low in comparison to the toothpastes either containing 5,000 ppm or 1,450 ppm F. Interestingly, the hardness measurements and mineral density were consistent in deionised water, 1,450 ppm, and 5,000 ppm F groups [17]. However, each technique used in that study had limitations in relation to the structural changes within the ARCLs according to formation of apatite using X-Ray diffraction analysis (XRD) following the use of different toothpastes.

In this current study, the XMT showed the increase of mineral density for all toothpaste groups during the 13 days pH-cycling in accordance with the previous study. In addition, the artificial root caries was used rather than natural one in this study. Artificial root caries without bacteria and enzymes could produce lesions most closely resembling natural lesions. Although the artificial root caries is a dead sample, the similarities between artificial and natural root caries in the demineralising system may indicate that this is not necessarily a major drawback in attempts to obtain a working model [27]. Qi et al., (2021) used the similar composition pH-cycling model for 14 days to prepare the artificial occlusal carious lesions by XMT assessment [22]. The authors reported the mineral loss with an approximately 0.3 mm thickness from surface to subsurface, which was similar mineral loss in this study by 5 days at the pH 4.8 demineralisation. As remineralisation involving in the pH-cycling model increases the hardness and mineral density, the demineralisation rate can be reduced. Therefore, pH 4.8 demineralisation solution only is recommended to form ARCLs.

As fluoride is a well-known remineralising agent, it can be speculated that this could be related to the acid resistant and less reactive fluorapatite layer [28]. This might suggest the precipitation of apatite mineral onto the ARCL surface as a result of hydroxyl ions being substituted (at least partly) by fluoride. This could explain the formation of either fluoride-substituted apatite or fluorapatite. The ^19^F-MAS-NMR signal between − 101.0 and − 107.0 ppm was used to identify the fluoride environment. The − 105.3 ppm chemical shift in the Group 1 indicated the origin of fluoride from the tooth itself as this tooth did not receive any fluoride application.

Gao et al., (2016) showed that ^19^F-MAS-NMR chemical shift plotted against the F percentage in the apatite. The 100% highly fluoridated apatite (fluorapatite) presents at -103.5 ppm chemical shift, whilst − 105.0 ppm would indicate a 40% fluoridated apatite [29]. The ^19^F-MAS-NMR spectra obtained for Group 2 at -105.1 ppm represented as fluoridated apatite. However, the formation of CaF_2_ would not be expected with toothpaste containing low fluoride as seen in the Group 2. In this study, the slight shoulder at -108.6 ppm seems to be an artefact. It can also be speculated that this might be formed following the use of toothpaste containing high fluoride *prior* to tooth extraction. In this respect, Chen et al., (2023) reported that the concentration of fluoride was less than 10 ppm after the toothpaste treatment with rinsing [17]. However, the slurries were diluted with water using the toothpastes containing 5,000 ppm F, 1,450 ppm F and BG with 540 ppm F and rinsed out after treatment in this present study. In addition, Mohammed et al., (2013) indicated the formation of CaF_2_ at over 45 ppm fluoride concentration. The presence of CaF_2_ has also been reported to exhibit anti-caries effect by forming a physical barrier on the enamel surface, thereby slowing the demineralisation process, and serving as a reservoir for fluoride [30, 31].

The 1,450 ppm and 5,000 ppm F toothpastes have intense peaks at -103 ppm. Mohammed et al., (2013) reported that the ^19^F MAS-NMR presented a sharp peak when enamel block is immersed in 11 ppm F [32]. The fluoride concentration for these two toothpastes in this study after rinsing was around 1–2 ppm [17], which resulted in fluorapatite as the major chemical species at low fluoride concentrations. Low fluoride levels found in saliva can significantly reduce enamel demineralisation, and those found in dental plaque fluid have a potential remineralisation effect [33].

Ekstrand et al., (2013) previously reported that the effect of 5,000 ppm fluoridated toothpaste was significantly more effective for arresting root carious lesion progression and promoting remineralisation compared to the 1,450 ppm fluoride toothpaste [7]. The mean numbers of hard lesions were 2.13 (1.68) in the 5,000 ppm fluoride toothpaste and 0.61 (1.76) in the 1,450 ppm fluoride toothpaste (*p* < 0.001). This current laboratory-based study also indicated the high mineral density in the toothpaste containing 5,000 ppm fluoride when compared to the 1,450 ppm one.

The present study demonstrated a decrease of mineral density in the subsurface for all toothpaste samples (Fig. 2). Previously, Ten Cate and Arends (1981) reported the blockage of surface layer pores as a result of fluoride-enhanced deposition within the surface [34]. Farooq et al., (2015) reported that BG toothpaste can occlude the dentinal tubules after simulated tooth brushing using SEM [35]. Once the remineralisation occurred at the beginning of pH-cycling, the pores of root dentine surface might have been obliterated. Therefore, the subsurface lesions would have been failed to remineralise further, since the formation of fluoride-substituted apatite was unable to pass through the surface to reach the subsurface. This was supported by the BSE images for high minerals at the edge of dentinal tubules however not within the dentine tubules. However, the SEM and BSE images also showed that few irregular white particles were noticeable on the subsurface of each sample rather than embedded in the dentinal tubules. It can be speculated that this could also be related to the polishing paste during the cutting and polishing process for the SEM analysis.

XMT was in 15 × 15 × 15 µm^3^ voxels, avoiding the errors in determining the sample thickness. In this respect, Davis et al., (2018) reported the XMT could be a useful method to evaluate demineralisation and remineralisation [36]. However, the XMT is unable to distinguish the mineral element to investigate the density change(s). The ^19^F MAS-NMR can indicate the formation of fluoridated apatite or fluorapatite, however the technique only analyses the powdered samples. It should be noted that each technique used in this study demonstrated some limitations, however they also complemented and supported each other with respect to the mineral density changes and fluorapatite formation. However, the system fails to distinguish the position of fluorapatite formation between the surface and subsurface. In addition, this study compared the SEM images for each group, however these images were not recorded at baseline and after the development of ARCLs due to the destructive nature of this technique. Theoretically, these samples would also have been exposed to fluoride environment in the oral cavity *prior* to extraction, which might have caused the variation of fluoride ions within the study teeth.

The small sample size could another limitation of this study. It should be noted that the samples are their own controls reducing the need for a large sample size required to account for variability in baseline mineral concentration since the XMT scanner was set at 15 μm voxel size/resolution (3D) for 24 h. The carious lesions for each sample were approximately 3 mm x 4 mm, therefore these high-resolution images can capture the full range of pore sizes in a representative elementary volume for the lesions. This unique XMT employed time-delay integration (TDI) to avoid ring artefacts and facilitate high signal-to-noise ratio imaging. Conventionally Transverse Microradiography (TMR) would have be used for quantifying the mineral density, however this technique requires a 100 μm thin tooth section. In addition, the grinding samples can produce undefined artefacts at the demineralised areas. The TMR technique involves the destruction of samples and scans of the same section which were unable to be detected before and after the intervention.

In addition, the XMT results were based on hundreds of slices to reconstruct the 3D images. This system can measure the same sample from the same slice at the start and end of the study which would provide a real comparison. The line scans were representative of the hundreds of slices. However, the variation in the mineral content of the teeth before the experiment would not have been seen in a conventional TMR study. Zain et al., (2020) also provided the evidence for the mineral changes in demineralised dentine for two teeth only using the same non-destructive 3D-XMT [37]. In conclusion, the TDI XMT takes 10 times longer than the conventional XMT, which would reduce the number of teeth, that can be evaluated.

In future laboratory-based studies, unerupted extracted wisdom teeth without any exposure or extracted teeth with root caries can be considered to mimic real life situation. In addition, the ^19^F-MAS-NMR might be used to assess the fluoride levels. Studies conducted without rinsing would also be interesting, since rinsing following tooth brushing is not recommended for the fluoride retention [17].

## Conclusion

Within the limitation of this laboratory-based study, all toothpastes were potentially effective to increase mineral density of artificial root caries on the surface, however there was evidence of mineral loss within the subsurface for Group 1, Group 3 and Group 4.

## Data Availability

The datasets generated and/or analysed during the current study are available from the first author upon request.
